# Development and Validation of a Digitizer-Based TCSPC System for Scintillation Decay Time Analysis via an Extended Convolution Model

**DOI:** 10.3390/s26051488

**Published:** 2026-02-27

**Authors:** Qianqian Zhou, Zhijie Yang, Wenhui Li, Juncheng Liang, Wuyun Xiao

**Affiliations:** 1 State Key Laboratory of Chemistry for NBC Hazards Protection, Beijing 102205, China; zhouqqstudio@163.com (Q.Z.); liwh1410@163.com (W.L.); 2 National Institute of Metrology, Beijing 100029, China; yangzhijie@nim.ac.cn

**Keywords:** time-correlated single-photon counting (TCSPC), deconvolution, instrument response function (IRF), digital twin, detector calibration

## Abstract

The development of high-fidelity digital twins for scintillation spectrometer detectors demands precise experimental characterization of timing parameters. This work presents a comprehensive solution comprising a digitizer-based time-correlated single-photon counting (TCSPC) system and an extended convolution model for decay time analysis. We introduce a physics-driven calibration principle, validating the system response against an independent physical benchmark to ensure fidelity. The proposed convolution model advances beyond the conventional model by incorporating additional parameters to account for scintillator-induced timing broadening and delay, thereby decoupling this effect from instrumental response. The model’s descriptive power was statistically validated through its application to fast scintillators, while its physical accuracy was robustly confirmed through the precise extraction of typical decay times from slow scintillators. This methodology establishes a reliable workflow from measurement to parameterization, directly supplying the decoupled inputs required for the digital twins of scintillation detectors.

## 1. Introduction

Precise characterization of the scintillation temporal response is fundamental to advancing radiation detection technologies, supporting applications including particle identification, high-resolution timing, and spectral unfolding. This need becomes increasingly critical with the emerging paradigm of digital twins for nuclear instruments, as high-fidelity virtual replicas of detectors require accurately validated physical parameters as input.

However, achieving such precise characterization is challenging, as the measured pulse is invariably a convolution of the intrinsic scintillation response and the instrument response function (IRF) of the detection system [1,2,3,4]. This IRF encompasses temporal broadening introduced by the photodetector and acquisition electronics [5]. Consequently, accurate reconstruction of the underlying scintillation kinetics requires both precise system characterization and effective deconvolution.

Time-correlated single-photon counting (TCSPC) is the standard technique for such timing characterization [6,7,8]. Most advanced TCSPC systems are designed to pursue ultimate timing resolution, achieving an IRF with a full width at half maximum (FWHM) below 100 ps by employing fast silicon photomultipliers (SiPMs) [7,9] paired with dedicated time-to-amplitude converters (TACs) or time-to-digital converters (TDCs). Although these systems provide excellent single-photon timing resolution, their effectiveness in high-fidelity pulse shape recovery is constrained by device-specific non-idealities, including afterpulse and optical crosstalk in SiPMs [10,11]. Such non-idealities introduce additional structure into the IRF and can degrade the accuracy of subsequent deconvolution. This presents a challenge for metrology-oriented applications, such as digital twin calibration, where a stable and fully characterized system response is often more valuable than raw picosecond resolution.

To address the need for a calibration-grade tool, we present a TCSPC methodology that prioritizes system characterization fidelity over ultimate timing resolution. This approach is based on the detection system employing photomultiplier tubes (PMTs), combined with high-speed waveform digitization and software-based analysis. This digitizer-based architecture simplifies the setup while providing direct access to waveforms for offline analysis. The core contribution is an extended convolutional model that explicitly parameterizes the timing broadening introduced by scintillators, which is a crucial step toward obtaining the decoupled inputs required for detector-scale digital twins.

## 2. Materials and Methods

### 2.1. Overview of the Methodology

To obtain decoupled detector parameters within a practical measurement and analysis procedure, we begin with the fundamental framework of TCSPC and its associated convolution model.

The scintillation pulse shape results from a multi-stage cascade: energy deposition, carrier transport, light emission at activator centers, and photon transport to the photodetector [12]. The TCSPC technique records this process by building a histogram of the time delay between a reference excitation signal and the subsequent detection of individual scintillation photons [13,14]. This measured distribution is inherently broadened by the finite timing resolution of the detection system. The fundamental relationship is formalized as [1]:

(1) $$f_{g}^{P}\left(t|\theta \right)=f^{P}\left(t|\theta \right)\;*\;H_{sys}(t).$$

In this model, $H_{sys}(t)$ denotes the system’s IRF and is given by Equation (2). The IRF is represented by a Gaussian function with a standard deviation $\sigma_{IRF}$ and an electronic delay ${\Delta t}_{IRF}$. $f^{P}\left(t|\theta \right)$ is the photon emission rate detected by the TCSPC system. In Equation (3), $f^{P}\left(t|\theta \right)$ comprises the intrinsic scintillation emission rate $f\left(t|\theta \right)$ and a prompt emission component as a Dirac-delta function δ(θ) with amplitude Camp. $f\left(t|\theta \right)$ is typically modeled as a multi-exponential function that includes both rise ($\tau_{r,i}$) and decay ($\tau_{d,i}$) times, with the process starting at time θ, as stated in Equation (4). The analytical convolution of these components yields the complete model for the measured data $f_{g}^{P}\left(t|\theta \right)$.

(2) $$H_{sys}(t)=\frac{1}{\sqrt{2\pi }\sigma_{IRF}}exp\left(-\frac{{\left(t-{\Delta t}_{IRF}\right)}^{2}}{2{\left(\sigma_{IRF}\right)}^{2}}\right)$$

(3) $$f^{P}\left(t|\theta \right)=f\left(t|\theta \right)+Camp\cdot \delta (\theta )$$

(4) $$f\left(t|\theta \right)=\sum_{i=1}^{N}\frac{\rho_{i}}{\tau_{d,i}-\tau_{r,i}}\left(e^{-(t-\theta )/\tau_{d,i}}-e^{-(t-\theta )/\tau_{r,i}}\right)$$

Therefore, precise recovery of the intrinsic scintillation decay kinetics encoded in $f\left(t|\theta \right)$ requires not only high-accuracy measurement of $f_{g}^{P}\left(t|\theta \right)$ but also independent and precise characterization of $H_{sys}(t)$ to enable effective numerical deconvolution.

### 2.2. Digitizer-Based TCSPC System

**Figure 1:**
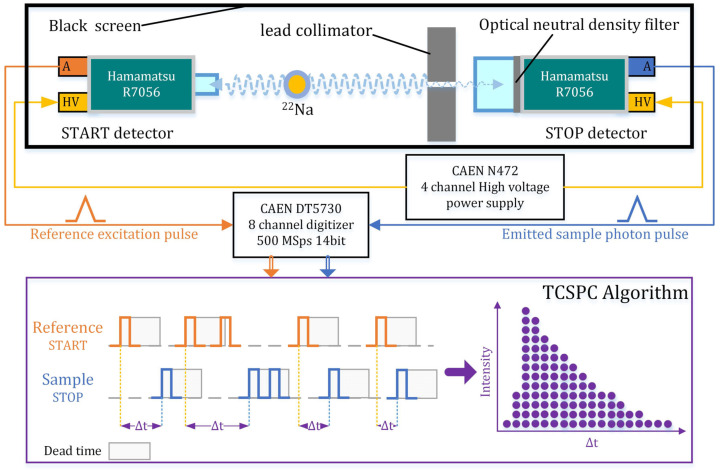
Schematic of the digitizer-based TCSPC setup.

The custom TCSPC system developed in this work (Figure 1) is designed to achieve high-precision temporal characterization of the scintillation pulse, with particular emphasis on single-photon detection reliability and rigorous IRF calibration. The system design addresses three critical requirements: (1) picosecond-level synchronization between excitation and detection events, (2) a well-characterized IRF, and (3) controlled single-photon detection probability to avoid pulse pile-up.

#### 2.2.1. Picosecond-Level Synchronization via Positron Annihilation Source

To establish a stable and physically inherent timing reference, a ^22^Na positron annihilation point source was employed. The time-correlated excitation is provided by coincident 511 keV γ-photon pairs, which are emitted in opposite directions (180°) with negligible temporal broadening on the picosecond scale [15]. This inherent back-to-back emission geometry provides the ideal excitation timestamp, meeting the stringent synchronization specified in requirement (1). Two identical detector channels were arranged symmetrically around the source to implement this coincidence timing. In this configuration, two R7056 PMTs (Hamamatsu, Japan), each with a typical rise time of 1.7 ns and a transit time spread (TTS) of 500 ps FWHM, were assigned to the START and STOP channels, respectively.

#### 2.2.2. IRF Characterization with an Optimized Reference Detector

A well-characterized system IRF is fundamental to the deconvolution process, corresponding to requirement (2). Accurate IRF measurement must capture the total jitter of the entire system, including both the START and STOP channels.

- Reference (START) detector optimization

The overall IRF width is fundamentally limited by the timing precision of the START signal. A comparative study of candidate radiators was conducted, including a Cherenkov radiator (H-K9L glass), BaF_2_:Y, and LYSO:Ce. LYSO:Ce was selected as the START detector based on its combination of high light yield (~32,000 photons/MeV) and fast decay (~40 ns), which provides a high signal-to-noise ratio (SNR) for excitation timing [16]. This choice yields a statistically robust and sharp trigger signal, minimizing the contribution of the START channel to the overall system jitter.

- IRF calibration procedure

The total system IRF was measured by coupling an LYSO:Ce crystal (CETC Chip Technology Co., Ltd., Chongqing, China) to the START channel and a prompt Cherenkov radiator (H-K9L glass, Nanyang Jingliang Optoelectronic Technology Co., Ltd., Nanyang, China) to the STOP channel. The Cherenkov radiator provides picosecond-level single-photon time resolution, which is negligible compared to the timing jitter of photodetectors and electronics [17]. Consequently, the measured histograms of the coincidence signals directly represent $H_{sys}(t)$ of the entire system. This $H_{sys}(t)$ encompasses the temporal broadening introduced by the LYSO:Ce scintillation process, the TTS of both PMTs, and the electronic jitter of the acquisition chain. This procedure yielded a system time resolution that serves as the essential kernel for subsequent deconvolution.

#### 2.2.3. Optimization of Single-Photon Detection Probability

As mentioned in requirement (3), TCSPC requires that the probability of detecting more than one photon per excitation event in the STOP channel be negligible [18]. This was achieved using the following approach:

- Photon flux attenuation

Optical neutral density (ND) filters were placed in direct contact with the photocathode of the STOP PMT, forming an integrated single-photon detection unit. The transmittance of these filters was selected based on the light yield of the scintillator under test to attenuate the photon flux, ensuring an average detection rate of well below one photon per trigger.

- Background suppression and collimation

A lead collimator was installed between the scintillator (or Cherenkov radiator) and the STOP single-photon detection unit, serving dual purposes. First, it shields the PMT window and ND filters from direct exposure to source γ-photons. This reduces the generation rate of parasitic Cherenkov light within these components, as Cherenkov radiation is a critical background source for low-light-yield scintillators. Additionally, this approach collimates the emitted light, preferentially selecting photons that propagate directly from the scintillator to the photocathode [13]. This further reduces the probability of multi-photon events caused by localized light scattering and helps establish a consistent optical path.

#### 2.2.4. Data Preprocessing and Histogram Construction

The anode signals from both PMTs were directly fed to a desktop fast digitizer, the CAEN DT5730 (CAEN SpA, Viareggio, Italy), which has a sampling rate of 500 MS/s and a resolution of 14 bits. The analog pulses were digitized and time-stamped using the digitizer’s firmware-based digital constant fraction discriminators (CFDs). The CFD implemented a 75% attenuation ratio, a 2 ns delay, and 2-point smoothing, achieving a fine time stamp resolution of 2 ps. Individual trigger thresholds were set according to charge-integrated pulse height spectra achieved by the digitizer pulse shape discrimination (PSD) algorithm. For the START channel, the threshold was placed above the electronic noise floor to select 511 keV full-energy events from the reference detector. For the STOP channel, the threshold was set as low as possible while still rejecting baseline noise, ensuring preferential detection of single-photon events. To eliminate overlapping events (pile-up) caused by high photon flux, the digitizer implemented a 1024 ns fixed dead time after each valid CFD trigger. Any secondary trigger detected within this window was flagged as pile-up and rejected. The remaining time-stamped events were then saved as a list sequence file containing the absolute arrival time of each detected event, which served as the raw data source for subsequent offline histogram generation of delay time.

The raw data acquired by the digitizer is a list-mode sequence of timestamps for START and STOP events. For each valid coincidence event, the time difference ΔT was calculated as $\Delta T=T_{stop}-T_{start}$. These time differences were then binned into a histogram to form the measured distribution $f_{data}$ of the scintillator sample. To balance time resolution and SNR, the original time bins (2 ps/bin) were merged into 200 ps/bin for samples, which improved fitting stability without distorting the intrinsic decay features. The same procedure with 30 ps/bin was applied to the data collected for the IRF calibration process to construct the $H_{sys}(t)$.

### 2.3. Extended Convolution Model and Parameter Extraction

With the experimentally acquired timing spectra $f_{data}$ and the characterized system response $H_{sys}(t)$ from Section 2.2, the next step is to extract the underlying physical parameters through the convolution model. Building on the foundational model framework outlined in Section 2.1, the conventional convolution model (Equation (7) in Reference [1]) unifies the temporal broadening parameter $\sigma_{IRF}$ and electronic delay ${\Delta t}_{IRF}$ for both scintillation photons and prompt photons. However, this simplification overlooks the intrinsic temporal signatures induced by photon propagation within the scintillator. These signatures are physically relevant for scintillation photons but can be disregarded for prompt photons like Cherenkov radiation. To address this limitation and improve the physical realism of the model, we extended the original formulation by separating the broadening parameters and delay times for scintillation and prompt photons, enabling explicit quantification of scintillator-specific temporal effects that are critical for digital twin-driven detector design.

#### 2.3.1. Extended Convolution Model

The extended model retains the original structure of the photon emission rate $f_{g}^{P}\left(t|\theta \right)$, combining multi-exponential scintillation emission and Dirac-delta prompt emission, and its convolution with the system IRF $H_{sys}(t)$. Key modifications are the separation of broadening and delay parameters for the two photon components, as expressed in Equation (5):

(5) $$\begin{array}{cc} f_{g}^{P,ext}\left(t|\theta \right) & =\sum_{i=1}^{N}\frac{\rho_{i}}{2\left(\tau_{d,i}-\tau_{r,i}\right)}exp\left(\frac{2\tau_{d,i}\left({\Delta t}_{s}+\theta -t\right)+\sigma_{s}^{2}}{2\tau_{d,i}^{2}}\right)\\ & \cdot \left(1-\mathrm{erf}\left[\frac{\tau_{d,i}\left({\Delta t}_{s}+\theta -t\right)+\sigma_{s}^{2}}{\sqrt{2}\sigma_{s}\tau_{d,i}}\right]\right)\\ & -\sum_{i=1}^{N}\frac{\rho_{i}}{2\left(\tau_{d,i}-\tau_{r,i}\right)}exp\left(\frac{2\tau_{r,i}\left({\Delta t}_{s}+\theta -t\right)+\sigma_{s}^{2}}{2\tau_{r,i}^{2}}\right)\\ & \cdot \left(1-\mathrm{erf}\left[\frac{\tau_{r,i}\left({\Delta t}_{s}+\theta -t\right)+\sigma_{s}^{2}}{\sqrt{2}\sigma_{s}\tau_{r,i}}\right]\right)\\ & +\frac{Camp}{\sqrt{2\pi }\sigma_{prompt}}exp\left(-\frac{{\left(t-\theta -{\Delta t}_{prompt}\right)}^{2}}{2\sigma_{prompt}^{2}}\right). \end{array}$$

Here, the original parameters retain their physical meanings, N denotes the number of exponential components; $\tau_{r,i}$ and $\tau_{d,i}$ are the rise and decay times of the i-th scintillation component; $\rho_{i}$ is the relative light yield weight ($\sum_{i=1}^{N}\rho_{i}=1$); θ is the positron annihilation time; Camp is the amplitude of the prompt emission component; and erf is the error function arising from the semi-analytical convolution solution [1].

The extended model introduces four new parameters to explicitly distinguish scintillator-induced effects from system response. For prompt photons, the temporal characteristics are dominated by the system’s IRF, with negligible contributions from photon transport time spread (PTS) or carrier dynamics. Thus, their broadening $\sigma_{prompt}$ and delay ${\Delta t}_{prompt}$ remain tightly coupled to the intrinsic system response. The temporal broadening $\sigma_{prompt}$ is equivalent to $\sigma_{IRF}$ due to negligible scintillator-induced effects. The delay time ${\Delta t}_{prompt}$ is dominated by electronic delay ${\Delta t}_{IRF}$, ${\Delta t}_{prompt}\approx {\Delta t}_{IRF}$ in practical fitting.

For scintillation photons, the temporal profile is shaped by two overlapping broadening mechanisms, system-induced instrument response $\sigma_{IRF}$ and scintillator-induced effects. The scintillator-induced effects include PTS from scattering or propagation path length variations and intrinsic carrier dynamics involving the process of energy transfer to activator centers [17,19]. These combined effects result in a total broadening $\sigma_{s}$, combining $\sigma_{IRF}$ and scintillator-specific broadening ($\sigma_{s,specific}=\sqrt{\sigma_{s}^{2}-\sigma_{IRF}^{2}}$). A physical constraint $\sigma_{s}\geq \sigma_{IRF}$ is imposed to reflect the additive nature of broadening mechanisms. Correspondingly, the delay time ${\Delta t}_{s}$ integrates both electronic delay ${\Delta t}_{IRF}$ and photon propagation time, which depends on crystal dimensions, wrapping material, and surface properties. This parameter quantifies the geometric- or surface-dependent delay, enabling independent optimization of material properties and detector packaging in predictive models.

#### 2.3.2. Parameter Extraction and Fitting Procedure

Having defined the extended convolution model in Equation (5), we established a statistically rigorous procedure to extract physical parameters from experimental TCSPC histograms and designed a strategy for validating the model.

The performance and validity of the extended convolution model were evaluated through a validation strategy designed to separately test its descriptive power and physical accuracy. First, the model’s capability to capture scintillator-induced effects was assessed by comparing its fit quality with that of the conventional model using fast scintillators (LYSO:Ce and LaBr_3_:Ce, Beijing Glass Research Institute Co., Ltd., Beijing, China), where such effects are most pronounced. Second, the model’s accuracy and robustness in extracting the fundamental decay constant were confirmed by applying it to well-characterized slow scintillators (BGO and NaI:Tl, Beijing Glass Research Institute Co., Ltd., Beijing, China) and comparing the extracted decay times with established literature values.

The model was fitted to the measured timing spectra via weighted χ^2^ (Equation (6)) minimization:

(6) $$\chi^{2}=\frac{1}{N_{bins}-N_{para}}\sum_{i=1}^{N_{bins}}{\left[{\left(f_{data}\right)}_{i}-{\left(f_{g}^{P,ext}\right)}_{i}\right]}^{2}/{\left(f_{g}^{P,ext}\right)}_{i},$$

where $N_{bins}$ denotes the total number of time bins used for fitting in the TCSPC histogram. $N_{para}$ is the number of free parameters in the extended model, accounting for the degrees of freedom lost during fitting. ${\left(f_{data}\right)}_{i}$ refers to the measured event count in the i-th time bin, while ${\left(f_{g}^{P,ext}\right)}_{i}$ is the corresponding model-predicted count from the extended convolution model. The weight ${\left(f_{g}^{P,ext}\right)}_{i}$ is the inverse of the predicted variance under Poisson statistics, providing an optimal approximation to the Poisson log-likelihood (Cash statistic) for high-count data. This formulation also enables the use of the weighted least squares Akaike information criterion (WLS-AIC) for statistically rigorous model comparison [20].

In the fitting procedure, the parameters of $H_{sys}(t)$ ($\sigma_{IRF}$ and ${\Delta t}_{IRF}$) were fixed to the values independently determined from the IRF measurement detailed in Section 2.2. The primary free parameters were the intrinsic scintillation decay time $\tau_{d,i}$ and the scintillator-induced broadening $\sigma_{s}$. Physically reasonable bounds were applied during minimization to ensure convergence to a realistic solution. The final output of this procedure for each scintillator sample was a set of decoupled parameters, including the material-specific decay constant $\tau_{d,i}$ and the sample-specific broadening parameter $\sigma_{s}$.

To quantify uncertainties arising from counting noise and the finite precision of the fixed IRF parameters, a parametric bootstrap was implemented. For each dataset, 1000 synthetic histograms were generated by Poisson resampling of the original data. The model was refitted to each synthetic dataset with the IRF parameters fixed. The standard deviation of the bootstrap replicates was used to estimate the uncertainties of each free parameter. All reported uncertainties were based on this procedure.

Together with the fixed system response $\sigma_{IRF}$, these parameters and their uncertainties constitute a physics-based characterization of the timing response, ready for integration into detector digital twin simulations.

## 3. Results

This section presents the performance characterization of the developed TCSPC system and the delay time spectrum analysis of four typical scintillators. All measurements were standardized at room temperature and ^22^Na excitation conditions to ensure data comparability.

### 3.1. Instrument Response Function Characterization

**Figure 2:**
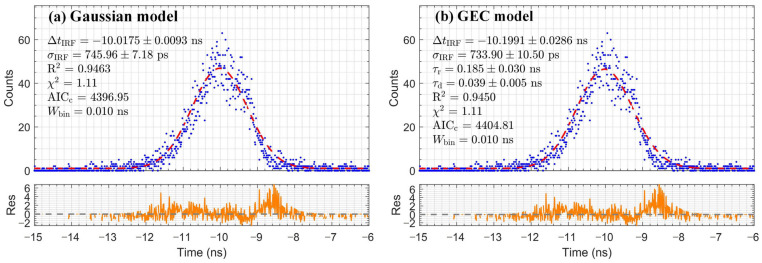
Delay time histogram data (blue dots) of the IRF and fitting results (red dashed line) with residuals (orange line) for (**a**) the Gaussian model and (**b**) the GEC model.

The IRF was measured under single-photon coincidence conditions to calibrate the temporal resolution limit of the TCSPC system and determine the IRF parameters for subsequent deconvolution. The measured IRF histogram is shown in Figure 2. Two analytical models were employed and compared: the Gaussian model, which describes symmetric temporal broadening dominated by electronic jitter, and the Gaussian double-exponential convolution (GEC) model [1], which can characterize the asymmetric tailing induced by the internal PMT processes. As shown in Figure 2, both models demonstrated good statistical agreement with the experimental data across the full time range, supported by R^2^ and reduced χ^2^ values close to 1. To quantitatively compare the models, we calculated the Akaike Information Criterion corrected for finite sample size (AICc). For the IRF fit, the Gaussian model yielded a marginally lower AICc (4397) than the GEC model (4405). However, this small difference (ΔAICc≈8) is insufficient for definitive model selection. Moreover, the significant difference in their extracted temporal parameters ($\Delta t_{IRF}$ and $\sigma_{IRF}$) required a physics-driven selection criterion.

**Figure 3:**
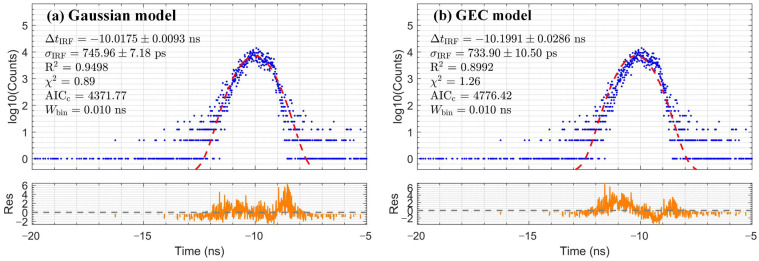
Comparison of fitting results (red dashed line) with residuals (orange line) for the BGO Cherenkov response data (blue dots), using IRF parameter sets from (**a**) the Gaussian model and (**b**) the GEC model.

Since the IRF was measured using Cherenkov radiation, we reasoned that the more physically accurate model should also optimally describe the Cherenkov light component isolated from scintillator measurements. Therefore, we evaluated the two candidate IRF parameter sets by fitting the BGO Cherenkov response, as shown in Figure 3. The fit using IRF parameters derived from the Gaussian model achieved a higher R^2^, a reduced χ^2^ close to 1, and a substantially lower AICc (4372) than the GEC-based fit (4776, ΔAICc≈404). This decisively identifies the Gaussian model as the more accurate representation of the system response under the same Cherenkov emission mechanism. Consequently, we adopted the Gaussian-derived parameters, including $\Delta t_{IRF}=-10.02\pm 0.01$ ns and $\sigma_{IRF}=745.96\pm 7.18$ ps, as the definitive IRF parameters. This corresponds to an IRF FWHM of approximately 1.75 ns. These parameters were used for all subsequent deconvolution analyses.

### 3.2. The Extended Convolution Model Validation

**Figure 4:**
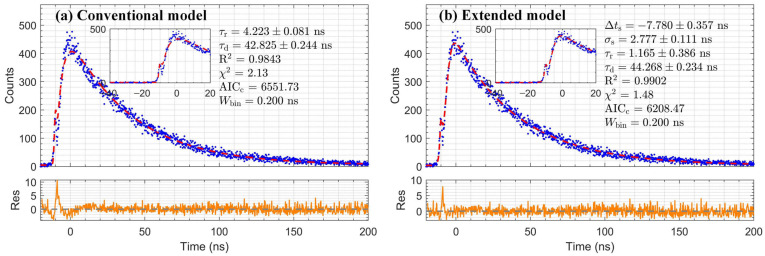
Comparison of fitting results (red dashed line) with residuals (orange line) for LYSO:Ce data (blue dots), using (**a**) the conventional model and (**b**) the extended model.

**Figure 5:**
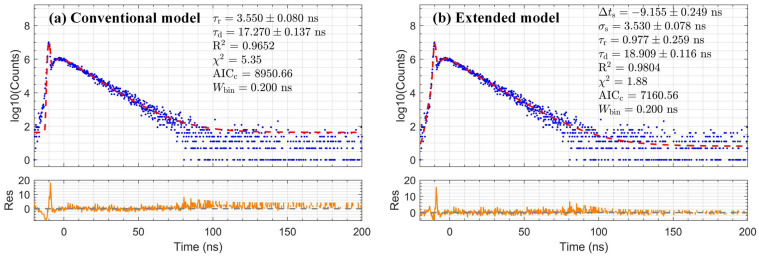
Comparison of fitting results (red dashed line) with residuals (orange line) for LaBr_3_:Ce data (blue dots), using (**a**) the conventional model and (**b**) the extended model.

Following the validation strategy outlined in Section 2.3, the performance of the extended convolution model was evaluated by applying it to the timing spectra of fast scintillators. Figure 4 and Figure 5 show fits to the LYSO:Ce and LaBr_3_:Ce data obtained with conventional and extended models, respectively. A quantitative comparison of the fitting metrics is summarized in Table 1. For both scintillators, the extended model consistently yields a higher R^2^, a reduced χ^2^ significantly closer to 1, and a substantially lower AICc than the conventional model. These statistical metrics confirm the extended convolution model is statistically superior to describe the experimental data. The decay times extracted by the extended model are 44.27 ± 0.23 ns for LYSO:Ce and 18.91 ± 0.12 ns for LaBr_3_:Ce.

### 3.3. Application of Slow Scintillators and Verification of Extracted Decay Time

**Figure 6:**
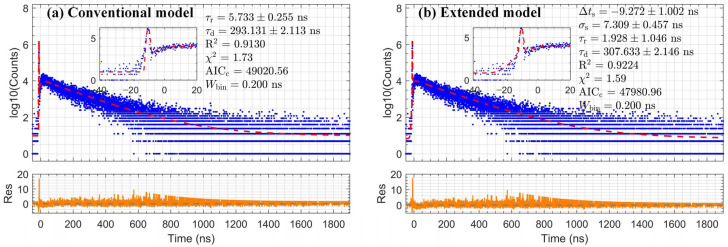
Comparison of fitting results (red dashed line) with residuals (orange line) for BGO data (blue dots), using (**a**) the conventional model and (**b**) the extended model.

**Figure 7:**
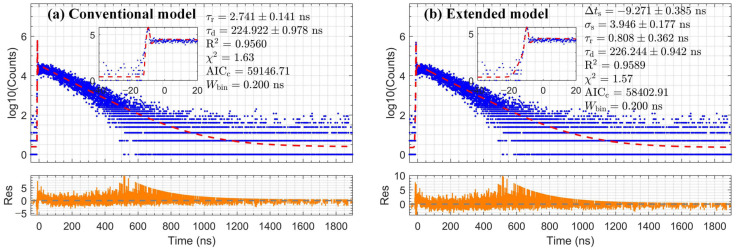
Comparison of fitting results (red dashed line) with residuals (orange line) for NaI:Tl data (blue dots), using (**a**) the conventional model and (**b**) the extended model.

Building on the validation of its performance in Section 3.2, the extended convolution model was applied to characterize two slow scintillators, BGO and NaI:Tl. For these scintillators, the extended and conventional models are expected to yield comparable fitting quality, as the long decay tail dominates the spectrum profile. Accordingly, this section focuses on evaluating the accuracy and reliability of the intrinsic decay time $\tau_{d}$ extracted using the extended model. The measured timing spectra for BGO and NaI:Tl and the optimal fits are presented in Figure 6 and Figure 7. The model accurately describes the data across the entire time range, as quantified by the fit metrics annotated in the figure. The decay times extracted for both scintillators are summarized in Table 2. The intrinsic decay times extracted for BGO and NaI:Tl are 307.63 ± 2.15 ns and 226.24 ± 0.94 ns, respectively. The extracted values show excellent consistency with widely cited literature values, approximately 300 ns for BGO and 230 ns for NaI:Tl [13].

## 4. Discussion

### 4.1. Physics-Driven Calibration Principle for TCSPC Systems

The model selection process in Section 3.1 exemplifies a transferable calibration principle for digitizer-based TCSPC systems: the optimal IRF must be validated not only by its mathematical fit to the measured IRF data, but also by its physical consistency with an independent benchmark sharing the same underlying photon detection mechanism.

Statistical metrics such as R^2^ and reduced χ^2^, while essential for assessing fit quality, are often insufficient to resolve model ambiguity when multiple functional forms describe the direct IRF measurement equally well. In such cases, the proposed physics-driven benchmark provides an independent and physically meaningful criterion to resolve this ambiguity. This demonstrates that the appropriate IRF model is not universal, but must be empirically determined for each setup using a validation framework grounded in the actual detection physics.

By validating candidate IRF parameters against an independent Cherenkov light benchmark derived from an actual scintillator, this approach ensures that the selected model faithfully represents the intrinsic system timing distribution. This physical validation step reduces the risk of mathematical misspecification and provides a more robust foundation for extracting intrinsic scintillation response, which is crucial for generating reliable input for digital twins of spectrometer detectors.

### 4.2. Performance and Applicability of the Extended Convolution Model

The statistically superior performance of the extended convolution model is demonstrated by its enhanced fit metrics for fast scintillators LYSO:Ce and LaBr_3_:Ce, as summarized in Section 3.2. The choice of fast scintillators for this validation is grounded in their timing characteristics. With intrinsic decay times on the order of tens of nanoseconds, the overall profile of their timing spectra is dominated by the rising edge and early decay period. This region is where contributions from the instrument response and the additional timing broadening introduced by the scintillator itself have a significant impact. Consequently, any deficiency of the model in representing these physical processes will be magnified in the overall fit quality for fast scintillators. The statistically superior fit provided by the extended model demonstrates that its additional parameters effectively capture this scintillator-induced broadening, which encompasses effects such as photon transport (due to size and packaging) and intrinsic carrier dynamics, neglected by the conventional model.

This capability represents a critical advancement for the physical parameterization within a digital twin framework. Rather than merely extracting a material decay constant, the model provides a separate parameterization of the scintillator’s contribution to timing broadening. The distinction between the intrinsic material property and the scintillator-induced broadening is crucial. It facilitates the provision of essential, independent inputs for predictive simulations of timing across various physical detector configurations.

In contrast, for slow scintillators, the spectrum is dominated by the long decay tail, making the fit less sensitive to refinements in modeling the early decay period. The application of the model to BGO and NaI:Tl therefore serves to validate its robustness and practical applicability. Strong agreement between our results and well-established literature values confirms the robustness and physical validity of the model. It also demonstrates that the model introduces no bias or instability when applied to scintillators where additional parameters are not crucial. Instead, it converges to physically consistent parameters, particularly the intrinsic decay time. This fulfills its role as a precise and reliable characterization tool within the digital twin framework.

### 4.3. Limitations and Future Work

The methodology presented here has been validated across a representative range of scintillators, but it is bounded by its current implementation. The primary limitation arises from the timing resolution determined by the use of PMTs. This choice was made to align with the primary objective of characterizing conventional scintillation spectrometer detectors, which predominantly employ PMTs. Consequently, the system replicates the timing performance of practical detectors, ensuring the extracted parameters are directly applicable to their digital twins. However, this limits the precise characterization of sub-nanosecond properties, such as the rise times or the detailed shapes of certain ultrafast scintillators.

These limitations provide clear directions for future work. The modular design of the digitizer-based TCSPC system makes it inherently adaptable. Upgrading to faster photoelectric sensors, such as SiPMs, can improve timing resolution [9,21,22]. This would enable the precise study of ultrafast scintillation processes and provide access to previously inaccessible parameters like intrinsic rise times. Such an upgrade would expand the range of scintillator materials available for high-fidelity digital models. The transition from PMT to SiPM-based TCSPC can be achieved with minimal modification to the current deconvolution framework. Crucially, under the single-photon counting regime inherent to TCSPC, the effects of optical crosstalk in SiPMs are negligible. Furthermore, afterpulse events can be effectively rejected by employing a suitable dead time longer than the characteristic release time of trapped carriers. Consequently, the primary impact of switching to a SiPM is reflected in the system’s instrumental response function $H_{sys}(t)$. The $H_{sys,SiPM}(t)$ would be measured following the same procedure outlined in Section 2.2, simply by replacing the PMT with the SiPM coupled to the Cherenkov radiator. This measured IRF inherently incorporates the single-photon time resolution and residual jitter of the SiPM under controlled, low-noise operating conditions. Therefore, the existing deconvolution model remains directly applicable, with the improved timing performance of the SiPM captured within the updated $H_{sys,SiPM}(t)$.

Additionally, the ability to decouple the intrinsic decay constant from the photon transport broadening provides essential inputs for detector-scale simulations. A key direction of our future work is to integrate these parameters into a full Monte Carlo radiation transport and optical photon simulation using Geant4 [23]. This framework will model the complete detector geometry, including crystal size, surface finish, wrapping, and optical coupling, and simulate the generation, propagation, and detection of scintillation photons. The predictive power of the digital twin will be validated by directly comparing the simulated timing spectrum with the experimental measurement obtained from the same detector configuration. Agreement in decay time, rise time, and overall spectral shape will serve as a quantitative benchmark for model fidelity. This step will transform the presented framework from a characterization tool to a core component of predictive engineering for scintillation spectrometers.

## 5. Conclusions

This study established and validated a coherent methodology for scintillator characterization, incorporating a flexible digitizer-based TCSPC system and a physics-enhanced analytical model. This work proposed a validated workflow that converts raw timing spectra into reliable parameters required for detector digital twins. The key component of this workflow is a calibration principle based on physical consistency, which ensures the accuracy of the system response. Furthermore, the developed convolution model effectively isolates the intrinsic scintillation decay dynamics from additional timing broadening induced by the scintillator. This distinction is critical for simulating detectors of different geometries and configurations. Validation across scintillators with disparate kinetics confirms that the model achieves improved fidelity in resolving scintillator-induced temporal broadening while maintaining robust accuracy in extracting standard decay times. Consequently, this work not only presents an enhanced measurement technique but also offers a foundational toolset to connect high-precision experimental data with the modeling frameworks employed in digital twins.

## Figures and Tables

**Table 1:** Comparison of model performance for LYSO:Ce and LaBr_3_:Ce.

Scintillator	Conventional Model				Extended Model				ΔAICc	Typical Range
	$\tau_{d}$ (ns)	R^2^	χ^2^	AICc	$\tau_{d}$ (ns)	R^2^	χ^2^	AICc		$\tau_{d}$ (ns)
LYSO:Ce	42.83 ± 0.24	0.9843	2.13	6552	44.27 ± 0.23	0.9902	1.48	6208	344	40–45
LaBr_3_:Ce	17.27 ± 0.14	0.9652	5.35	8951	18.91 ± 0.12	0.9804	1.88	7161	1790	15–20

**Table 2:** Validation of extracted decay times for BGO and NaI:Tl.

Scintillator	Conventional Model				Extended Model				ΔAICc	Typical Value
	$\tau_{d}$ (ns)	R^2^	χ^2^	AICc	$\tau_{d}$ (ns)	R^2^	χ^2^	AICc		$\tau_{d}$ (ns)
BGO	293.13 ± 2.11	0.9130	1.73	49,021	307.63 ± 2.15	0.9224	1.59	47,981	1040	300
NaI:Tl	224.92 ± 0.98	0.9560	1.63	59,147	226.24 ± 0.94	0.9589	1.57	58,403	744	230

## Data Availability

The data presented in this study are available on request from the corresponding authors.

## Abbreviations

The following abbreviations are used in this manuscript:

CFD	constant fraction discriminators
FWHM	full width at half maximum
GEC	Gaussian double-exponential convolution
IRF	instrument response function
ND	neutral density
PMT	photomultiplier tube
PTS	transport time spread
SiPM	silicon photomultiplier
SNR	signal-to-noise ratio
TCSPC	time-correlated single-photon counting
TTS	transit time spread

## References

[1] Gundacker S., Turtos R.M., Auffray E., Lecoq P. (2018). Precise Rise and Decay Time Measurements of Inorganic Scintillators by Means of X-Ray and 511 keV Excitation. Nucl. Instrum. Methods Phys. Res. Sect. Accel. Spectrometers Detect. Assoc. Equip..

[2] Gundacker S., Auffray E., Pauwels K., Lecoq P. (2016). Measurement of Intrinsic Rise Times for Various L(Y)SO and LuAG Scintillators with a General Study of Prompt Photons to Achieve 10 Ps in TOF-PET. Phys. Med. Biol..

[3] Gundacker S., Auffray E., Jarron P., Meyer T., Lecoq P. (2015). On the Comparison of Analog and Digital SiPM Readout in Terms of Expected Timing Performance. Nucl. Instrum. Methods Phys. Res. Sect. Accel. Spectrometers Detect. Assoc. Equip..

[4] Herweg K., Rutstrom D., Nadig V., Stand L., Melcher C.L., Zhuravleva M., Schulz V., Gundacker S. (2024). Timing Limits of Ultrafast Cross-Luminescence Emission in CsZnCl-Based Crystals for TOF-CT and TOF-PET. EJNMMI Phys..

[5] Xiao W., Farsoni A.T., Yang H., Hamby D.H. (2014). A New Pulse Model for NaI(Tl) Detection Systems. Nucl. Instrum. Methods Phys. Res. Sect. Accel. Spectrometers Detect. Assoc. Equip..

[6] Bollinger L.M., Thomas G.E. (1961). Measurement of the Time Dependence of Scintillation Intensity by a Delayed-Coincidence Method. Rev. Sci. Instrum..

[7] Nadig V., Herweg K., Chou M.M.C., Lin J.W.C., Chin E., Li C.-A., Schulz V., Gundacker S. (2023). Timing Advances of Commercial Divalent-Ion Co-Doped LYSO:Ce and SiPMs in Sub-100 Ps Time-of-Flight Positron Emission Tomography. Phys. Med. Biol..

[8] Wahl M., Roehlicke T., Kulisch S., Rohilla S., Kraeamer B., Hocke A.C. (2020). Photon Arrival Time Tagging with Many Channels, Sub-Nanosecond Deadtime, Very High Throughput, and Fiber Optic Remote Synchronization. Rev. Sci. Instrum..

[9] Gundacker S., Turtos R.M., Auffray E., Paganoni M., Lecoq P. (2019). High-Frequency SiPM Readout Advances Measured Coincidence Time Resolution Limits in TOF-PET. Phys. Med. Biol..

[10] Acerbi F., Gundacker S. (2019). Understanding and Simulating SiPMs. Nucl. Instrum. Methods Phys. Res. Sect. Accel. Spectrometers Detect. Assoc. Equip..

[11] Rosado J., Aranda V.M., Blanco F., Arqueros F. (2015). Modeling Crosstalk and Afterpulsing in Silicon Photomultipliers. Nucl. Instrum. Methods Phys. Res. Sect. Accel. Spectrometers Detect. Assoc. Equip..

[12] Yanagida T. (2018). Inorganic Scintillating Materials and Scintillation Detectors. Proc. Jpn. Acad. Ser. B Phys. Biol. Sci..

[13] Moszyński M., Gresset C., Vacher J., Odru R. (1981). Timing Properties of BGO Scintillator. Nucl. Instrum. Methods Phys. Res..

[14] Seifert S., Steenbergen J.H.L., van Dam H.T., Schaart D.R. (2012). Accurate Measurement of the Rise and Decay Times of Fast Scintillators with Solid State Photon Counters. J. Instrum..

[15] Moszyński M., Gierlik M., Kapusta M., Nassalski A., Szczęśniak T., Fontaine C., Lavoute P. (2006). New Photonis XP20D0 Photomultiplier for Fast Timing in Nuclear Medicine. Nucl. Instrum. Methods Phys. Res. Sect. Accel. Spectrometers Detect. Assoc. Equip..

[16] Lecoq P., Fabjan C.W., Schopper H. (2020). Scintillation Detectors for Charged Particles and Photons. Particle Physics Reference Library: Volume 2: Detectors for Particles and Radiation.

[17] Brunner S.E., Gruber L., Marton J., Suzuki K., Hirtl A. (2014). Studies on the Cherenkov Effect for Improved Time Resolution of TOF-PET. IEEE Trans. Nucl. Sci..

[18] Moses W.W. (1993). A Method to Increase Optical Timing Spectra Measurement Rates Using a Multi-Hit TDC. Nucl. Instrum. Methods Phys. Res. Sect. Accel. Spectrometers Detect. Assoc. Equip..

[19] Gundacker S., Knapitsch A., Auffray E., Jarron P., Meyer T., Lecoq P. (2014). Time Resolution Deterioration with Increasing Crystal Length in a TOF-PET System. Nucl. Instrum. Methods Phys. Res. Sect. Accel. Spectrometers Detect. Assoc. Equip..

[20] Banks H.T., Joyner M.L. (2017). AIC under the Framework of Least Squares Estimation. Appl. Math. Lett..

[21] Caccia M., Nardo L., Santoro R., Schaffhauser D. (2019). Silicon Photomultipliers and SPAD Imagers in Biophotonics: Advances and Perspectives. Nucl. Instrum. Methods Phys. Res. Sect. Accel. Spectrometers Detect. Assoc. Equip..

[22] Jiang W., Chalich Y., Deen M.J. (2019). Sensors for Positron Emission Tomography Applications. Sensors.

[23] Ogawara R., Ishikawa M. (2016). Signal Pulse Emulation for Scintillation Detectors Using Geant4 Monte Carlo with Light Tracking Simulation. Rev. Sci. Instrum..

